# Comprehensive Analysis of Metabolome and Transcriptome Reveals the Regulatory Network of Coconut Nutrients

**DOI:** 10.3390/metabo13060683

**Published:** 2023-05-24

**Authors:** Hao Guo, Chun Li, Jun Lai, Haiyang Tong, Zhenfeng Cao, Chao Wang, Wenyu Zhao, Liqiang He, Shouchuang Wang, Jun Yang, Tuan Long

**Affiliations:** 1 Sanya Nanfan Research Institute, Hainan Yazhou Bay Seed Laboratory, Hainan University, Sanya 572025, China; 2 College of Tropical Crops, Hainan University, Haikou 570228, China

**Keywords:** coconut, metabolome, transcriptome, glutathione, polyamines, α-linolenate

## Abstract

Coconut flesh is widely consumed in the market for its good flavor. However, a comprehensive and dynamic assessment of the nutrients in coconut flesh and their molecular regulatory mechanisms is lacking. In this study, the metabolite accumulation and gene expression of three representative coconut cultivars belonging to two subspecies were investigated using ultra performance liquid chromatography/tandem mass spectrometry. A total of 6101 features were detected, of which 52, 8, and 158 were identified as amino acids and derivatives, polyamines, and lipids, respectively. The analysis of the metabolite pathway showed that glutathione and α-linolenate were the main differential metabolites. Transcriptome data revealed significant differences in the expression of five glutathione structural genes and thirteen polyamine-regulated genes, consistent with trends in metabolite accumulation. Weighted correlation network and co-expression analyses showed that a novel gene *WRKY28* was implicated in the regulation of lipid synthesis. These results broaden our understanding of coconut nutrition metabolism and provide new insights into the molecular basis of coconut nutrition metabolism.

## 1. Introduction

Coconut (*Cocos nucifera* L.) is a dioecious plant (2n = 2x = 32) with a haploid genome of approximately 2.4 Gb [1,2,3], which is native to the coastal regions of Melanesia and Southeast Asia [4]. According to plant morphology, coconuts can be divided into two subspecies: *Cn. tall* (*Cocos nucifera* tall) and *Cn. dwarf* (*Cocos nucifera* dwarf) [2,3]. *Cn. tall* plants are usually approximately 25 m tall, and the flesh is rich in fatty acids but low in amino acids. In contrast, *Cn. dwarf* plants are generally 5–15 m in height, and the flesh is relatively poor in fatty acids but rich in amino acids [5,6,7,8]. Due to its tolerance to stress, *Cn. tall* is often planted by the sea as a landscape plant. *Cn. dwarf*, on the other hand, is often used as a raw material for edible goods or snack preparations due to its good fruit flavor.

Amino acids, polyamines, and lipids are important nutrients for human health [8]. Amino acids are one of the products of plant primary metabolism and biosynthetic precursors of glutathione. Glutathione plays a key role in reducing oxidative stress, maintaining redox balance, enhancing metabolic detoxification, and regulating the immune system [9]. Polyamine is a secondary metabolite produced from ornithine and arginine. Humans are more likely to lower blood pressure and reduce the risk of cardiovascular disease after eating foods rich in spermidine [10,11,12]. In addition, compared to other vegetable oils, coconut is also rich in some unsaturated fatty acids, such as linolenic (C18:3) acid, which is one of the most important sources of unsaturated fatty acids for industry [5,13,14]. As an important fatty acid for the human body, linolenic acid has neuroprotective, anti-tumor, antioxidant, anti-inflammatory, and antihypertensive effects [15,16]. There is a lot of research on amino acids in coconut, but the nutritional importance of polyamines and lipids is less known.

Metabolome and transcriptome analyses are powerful tools for identifying new nutrients and exploring the molecular basis of metabolic pathways. A study on citrus using untargeted metabolomics showed that more than 2000 metabolite signals were detected, and 54 metabolites were identified, including amino acids, flavonoids, and limonoids, demonstrating that the accumulation patterns of flavonoids in different subgroups and tissues of citrus were different [17]. Through transcriptome analysis, metabolic analysis, and pathway reconstruction, eight genes related to *N*-formyl colchicine biosynthesis were identified from *Gloriosa superba*, which almost completely explained the pathway of colchicine biosynthesis [18]. Using metabolome and transcriptome methods to assess nutrition stress of coconut, a study revealed the key metabolic pathway of potassium deficiency in coconut seedlings [2,19].

Although a number of studies on coconut nutrients have been conducted [8], the molecular mechanisms behind the biosynthesis of amino acids, polyamines, and lipids in coconut are still unclear. In this study, by integrating multi-omics analysis, we identified a number of new nutrients and reconstructed and compared the expression matrix and biosynthesis pathways of glutathione, polyamine, and α-linolenate in the flesh of *Cn. tall* and *Cn. dwarf*. Our results reveal the different molecular mechanisms behind amino acids, polyamines, and lipids biosynthesis in the two subspecies and broaden our understanding of the nutritional quality of coconut.

## 2. Materials and Methods

### 2.1. Plant Materials and Sampling

Coconut fruits of three Chinese cultivars, HT, YD, and GD, were used to analyze metabolome and transcriptome. HT is a representative cultivar that belongs to a coconut subspecies *Cn. tall*, and YD and GD are representative cultivars that belong to another coconut subspecies *Cn. dwarf*. All coconut trees were untreated and maintained at the Coconut Research Institute of the Chinese Academy of Tropical Agricultural Sciences, Hainan, China. The trees were planted in the same plantation at the same time, ensuring consistency in environmental factors. Fruits from 8-year-old trees were collected after flowering for 8 months for analysis. Six fruits were collected per tree and mixed as one biological replicate, and a total of six trees per cultivar were harvested as six biological replicates. After the fruit was picked, they were removed from the shell with a spoon, chopped, and collected in a 50 mL polypropylene centrifuge tube in liquid nitrogen.

### 2.2. Sample Preparation and Extraction

The flesh tissue was ground with a stirrer with zirconia beads at 30 Hz for 30 s at a low temperature. A total of 100 mg powder was weighed and extracted with 1.0 mL 70% methanol aqueous solution at 4 °C for 10 h. After centrifugation at 10,000× *g* for 10 min at 4 °C, the supernatant was filtered (SCAA-104, 0.22 μm pore size; ANPEL, Shanghai, China) and analyzed with LC–MS [20,21].

### 2.3. Quadrupole-Orbitrap UHPLC–MS/MS Analysis

Ultra performance liquid chromatography/tandem mass spectrometry (UHPLC–MS/MS) analysis was performed on a Thermo Fisher Q Exactive Plus mass spectrometer (Waltham, MA, USA; Thermo, Bremen, Germany). The separation was performed on a Waters CORTECS T3 Column (2.7 μm, 2.1 mm × 100 mm). The temperature of the autosampler and the column was set at 10 °C and 40 °C, respectively. The gradient elution flow rate was 0.4 mL/min. The mobile phase was acidified water (0.04% acetic acid water solution, *v*/*v*) (mobile phase A) and acidified acetonitrile (0.04% acetic acid acetonitrile, *v*/*v*) (mobile phase B). The linear gradient of mobile phase B was 5–95% for 0–10 min, 95% for 10–11 min, 95–5% for 11–11.1 min, and 5% for 11.1–15 min. The 2 μL sample was injected into the system and analyzed in electrospray positive ion (ESI) mode. The mass spectrometer performed full MS and ddMS2 scans. The full MS scan optimized acquisition parameters were as follows: resolution 70,000 full width at half maximum (FWHM); Automatic Gain Control (AGC) target 3 × 10^−6^; maximum injection time 100 milliseconds (IT, the maximum time allowed to obtain the set AGC target); and scan range 120–1800 *m*/*z*. The DdMS2 scan optimized acquisition parameters were as follows: resolution 35,000 FWHM; AGC target 1 × 105; maximum IT 50 milliseconds; loop count 12 and MSX count 1 (TopN 12); isolation window in quadrupole 3 *m*/*z*; and specific normalized collision energy (NCE) for each precursor *m/z* in 20, 40, 60:Dynamic exclusion auto. During acquisition, the mass spectrum was collected with the Xcalibur 4.1 software (Thermo Fisher Scientific, San Jose, CA, USA) for metabolomic analysis.

### 2.4. Comprehensive Metabolomic Analysis of Coconut Fruit

All raw data were aligned, deconvoluted, and converted using Compound Discoverer [version 3.1]. The results were displayed in tables, and the retention time (RT), precursor ions (Q1), MS/MS, and relative intensity of the target metabolites were obtained for each data file in the table. The peak intensity of the metabolites was used for quantitative analysis. SIRIUS [version 5.54] identified the original signal together and further identified the metabolites with Finger ID and GNPS [22,23,24]. At the same time, the compound information was compared with LIPDMAPS to identify more accurate lipid metabolites [25].

### 2.5. Metabolome Data Analysis

The filtered data were submitted to R software (www.r-project.org (accessed on 20 January 2023)) for orthogonal partial least squares-discriminant analysis (OPLS-DA) with ropls package (https://bioconductor.org/packages/release/bioc/html/ropls.html (accessed on 20 January 2023). Hierarchical clustering analysis of the metabolites between the samples was performed using R packages. For identifying differentially accumulated metabolites (DAMs), a fold change ≥ 2 or a fold change ≤ 0.5 and variable importance in the project (VIP) ≥ 1 was used as the screening criteria.

### 2.6. RNA Sequencing and Data Analysis

Total RNA was extracted from frozen fruits, and messenger RNA (mRNA) libraries were constructed for each sample and sequenced using the Illumina HiSeq-2000 platform. For data analysis, paired reads were mapped to the *Cn. dwarf* genome assembly using HISAT 2 with default parameters [26]. FeatureCounts counted the mapped fragments for each gene, and transcripts per million (TPM) were calculated. Genes with averaged TPM (replicates = 3) were considered expressed [27]. Principal component analysis (PCA) was performed to compare the TPM values of the expressed gene profiles among the development and ripening stages using the FactoMineR and factoextra function in R. Hierarchical clustering and heatmaps of the expressed genes were generated using the PHEATMAP packages (https://cran.r-project.org/web/packages/pheatmap/pheatmap.pdf (accessed on 20 January 2023) in R. Based on the raw count data, differential expression analysis between samples was performed with DESeq2 software [28]. Genes satisfying |log2Fold Change| ≥ 1.5 and False Discovery Rate (FDR) < 0.05 were defined as differentially expressed genes (DEGs) and subjected to Kyoto Encyclopedia of Genes and Genomes (KEGG) and Gene Ontology (GO) enrichment analysis.

### 2.7. Weighted Correlation Network Analysis and Gene Network Visualization

The weighted gene co-expression network analysis (WGCNA) package in R (WGCNA version 1.70-3) was used to generate co-expression network modules after discarding undetectable or relative low expression genes (TPM < 1) [29,30]. The co-expression modules were obtained using an automatic network construction function (block wise Modules) with default parameters apart from the soft threshold power of 9, TOMtype was signed, mergeCutHeight was 0.15, and minModuleSize was 10. The genes are divided into different modules based on the expression patterns of other genes. Then, the relationship between the genes and coconut metabolic traits is further searched by calculating the module’s characteristic values. By extracting the structural genes and promoter sequences that may participate in the pathway in the module (2000 kb upstream of the transcription start site), combining the information provided by PlantCARE to screen the transcription factors in the module, and then calculating metabolites and genes as well as metabolites and metabolites and the correlation coefficient between genes and genes, we could further construct a transcriptional regulatory network. The networks were visualized with Cytoscape [31].

## 3. Results

### 3.1. Metabolome Profiling of Different Coconut Cultivars

**Figure 1:**
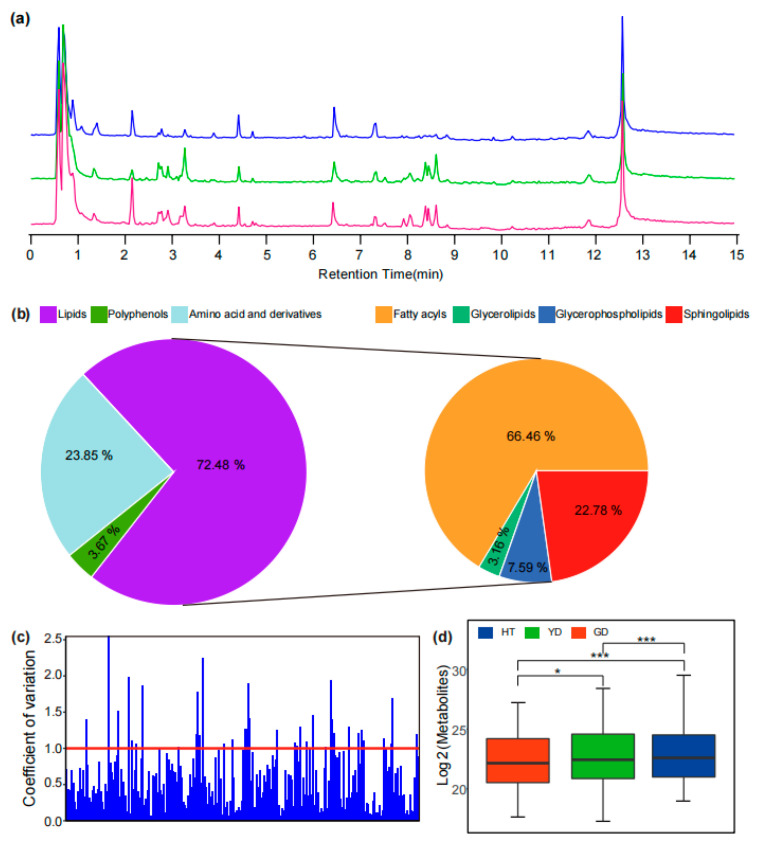
Detection and identification of lipids with UHPLC–HRMS. (**a**) TIC (Total ion chromatogram) of metabolites in different varieties of coconut obtained from UHPLC–MS/MS analysis in positive mode. The blue line represents HT, the green line represents YD, and the red line represents GD. (**b**) The classification of identified metabolites in coconuts includes amino acids and derivatives, polyamines, and lipids. (**c**) Analysis of the coefficient of variation of metabolites in coconuts. (**d**) Comparison of overall nutrient metabolite content in different cultivars. * and *** represent *p* < 0.05 and *p* < 0.001 by *t* tests, respectively.

To explore the metabolome variation of different coconut flesh, we selected three representative coconut cultivars for metabolome detection. A total of 6101 mass spectral signals were detected with Q Exactive-Orbitrap UHPLC–MS/MS in positive ion mode with untargeted scanning (Figure 1a). Through comparison, it was found that there were different metabolite accumulation patterns in the flesh of different coconuts. HT was higher than the other coconuts in terms of chromatographic peak number and peak height, while YD and GD were highly similar.

In order to elucidate the metabolome composition of coconut flesh, we utilized various strategies to analyze the metabolic product structure of the detected mass spectrometry signals. First, metabolites with commercial standards available were identified by comparing their retention time (RT), precursor ions (Q1), and MS/MS spectra with those of the commercial standard. For instance, a feature (Cm083) had an exact *m*/*z* value of 281.24564 for its precursor ion and a series of characteristic fragments of 263.23557 and 245.22447. Compared with the standard, the feature Cm083 was identified as linoleic acid due to the same mass spectral fragmentation information and RT (Figure S1a and Figure 1c). For metabolites without commercial standards, the mass spectrometry information obtained from the experiment was compared with published studies or metabolic databases to preliminarily resolve the metabolite structure. For instance, the exact *m*/*z* value of 520.33807 was detected in the MS1 spectrum of the MS signal (Cm204), and the [Y_0_]^+^ ion 184.07251 was observed in the MS/MS fragment, which was due to the characteristic fragment of phosphocholine; therefore, it was speculated that the mass spectrum signal Cm204 was LysoPC (18:2) (Figure S1b and Figure 1d). At the same time, the online metabolic databases, including mzCloud, LIPID MAPS, ChemSpider [23,26,32,33], and MassBank, were automatically matched in batches with the Compound Discoverer and SIRIUS for metabolite structure identification.

A total of 52 amino acids and derivatives, 8 polyamines, and 158 lipid metabolites were identified (Figure 1b and Table S1). The coefficient of variation (CV) can reflect the degree of dispersion of metabolites in the cultivars. Among the three cultivars, there were 36 metabolites with CV values greater than 1 (Figure 1c and Table S2), accounting for 50% of the total number of polyamines, which indicated that most of the polyamines differed greatly in the content of different cultivars of coconut flesh. Further, we performed OPLS-DA based on the identified metabolites to investigate the overall metabolome differences among the cultivars. The results showed that all six biological replicates of each cultivar clustered together followed by YD and GD, but both clustered further away from HT, which clustered separately (Figure S2). This result suggested that there may be a significant difference in the accumulation of these nutrients between *Cn. tall* and *Cn. dwarf* coconuts. In addition, the total nutrient metabolite content in the different cultivars was also different (Figure 1d). To further explore the differentially accumulated metabolites (DAMs), we used OPLS-DA for identification (See Method). The number of lipids in these DAMs is the highest reaching 100, of which 18 significantly differed in all comparisons (Figure S3 and Table S3). We further found that the DAMs were predominantly fatty acyl, accounting for 41.18%, followed by sphingolipids, accounting for 15.03% (Table S3).

### 3.2. Transcriptome Analysis of Different Coconut Cultivars

**Figure 2:**
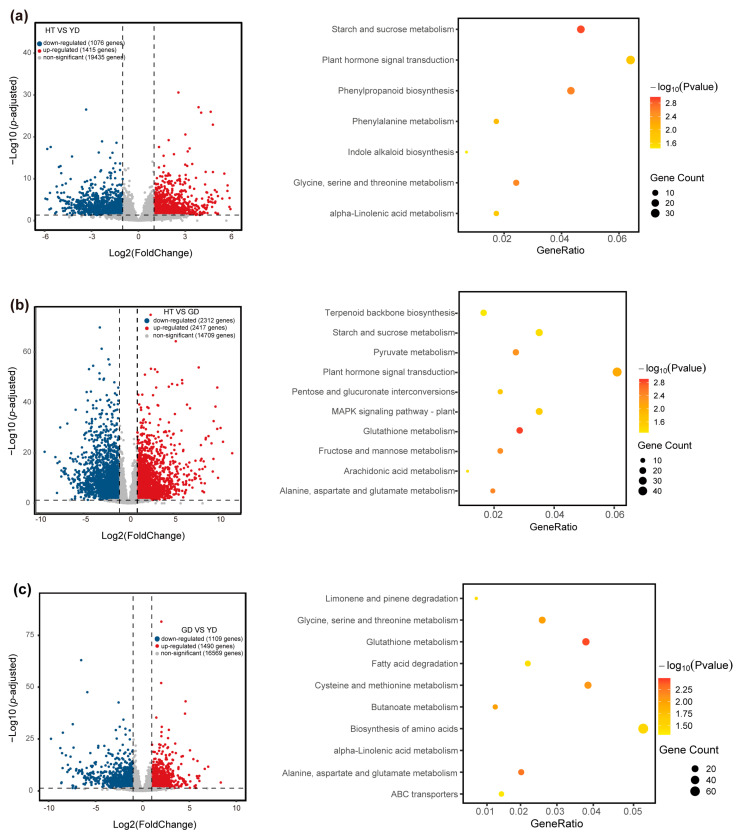
Comparative transcriptome analysis. The volcano plot and KEGG enrichment analysis of differentially expressed genes between HT and YD (**a**), HT and GD (**b**), and GD and YD (**c**).

To explore the variation of gene transcript levels among the coconut cultivars, we sequenced the transcriptome of coconut flesh tissue, obtaining a total of 58 Gb of 20,992 clean data (Table S4). Quantification of the transcriptome data revealed a total of genes expressed in at least one sample (TPM > 1) (Table S5). To understand the dynamics of transcript levels in the different coconut cultivars, we performed a principal component analysis (PCA) based on the transcriptome quantification results (Figure S4). The PCA result showed that samples of the same cultivars clustered together, whereas samples from different cultivars stayed away from each other. These results suggest that different cultivars exhibit different transcriptional level. To further determine the factors responsible for transcriptome differences, we performed differentially expressed genes (DEGs) analysis (See Methods). The results showed that 1456, 2909, and 1296 DEGs (YD vs. HT, GD vs. HT, GD vs. YD) were identified, respectively. A total of 3584 genes were differentially expressed in pairwise comparisons, of which 197 were core DEGs (Figure 2 and Tables S6–S8).

To explore the molecular function of the DEGs, KEGG enrichment analyses were performed. The results revealed that the DEGs between HT and YD were mainly related to starch and sucrose metabolism, phenylpropanoid biosynthesis, and glycine, serine, and threonine metabolism (Figure 2a). The DEGs between HT and GD were mainly related to glutathione metabolism, plant hormone signal transduction, and alanine, aspartate, and glutamate metabolism (Figure 2b). The DEGs between YD and GD were mainly related to α-linolenic acid metabolism, fatty acid degradation, and glutathione metabolism (Figure 2c). GO enrichment analysis showed similar results (Figures S6–S8). These results suggested that YD and GD differ greatly in the expression of fatty acid genes, and the DEGs between HT and YD and HT and GD were enriched in phenylpropanoid biosynthesis and amino acids such as glutathione metabolism. This also implied an association between variations of gene expression and metabolite content.

### 3.3. Analysis of Glutathione Metabolic Pathways in Coconuts

**Figure 3:**
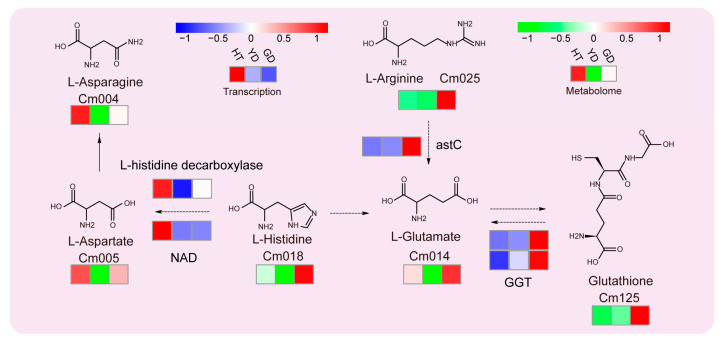
Transcriptome and metabolome analysis of the glutathione metabolic pathway. The heatmap represents the change in the expression levels of the genes and the accumulation levels of the metabolites. From red to blue or to green, the heatmap indicates the expression levels of genes or the accumulation levels of metabolites ranging from high to low. From left to right, HT, YD, and GD cultivars are shown.

Since glutathione metabolism was the most significantly enriched in the KEGG results (Figure 2), we analyzed this metabolic pathway in depth. All six metabolites located in this pathway were DAMs. L-asparagine and L-aspartate showed the highest accumulation in HT followed by GD. The other four metabolites, including L-histidine, L-arginine, L-glutamate, and L-arginine, showed opposite results, especially glutathione, which accumulated much higher in GD than HT and YD (Figure 3). Combined with the transcriptome expression matrix, we explored the expression levels of the related genes in this metabolic pathway (Figure 3). Both the L-histidine decarboxylase gene and *NAD*, the L-aspartate biosynthesis gene, exhibited extremely high expression levels in HT. In contrast, glutathione biosynthesis genes, such as *astC* and *GGT*, were shown to be highly expressed in GD. The gene expression was consistent with the level of metabolite accumulation, suggesting that variations in gene expression lead to differential accumulation of glutathione.

### 3.4. Regulatory Mechanisms of Differential Polyamine Metabolism

**Figure 4:**
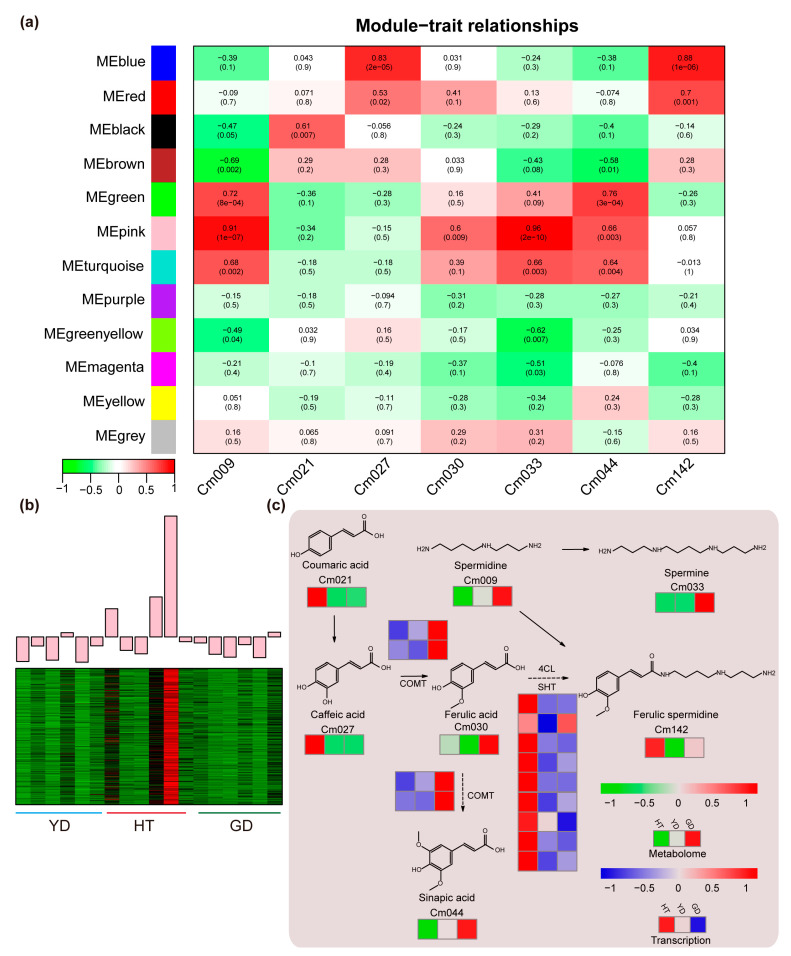
WGCNA and pathway analysis of the polyamine metabolic pathway. (**a**) Heatmap of the correlation between module genes and metabolites: each row represents a module, each column represents a metabolite, and the color and value of each cell at the intersection of the row and column represent the correlation coefficient between the module and the sample and *p*-value. (**b**) Expression profiles of “MEpink” modules in different coconut cultivars. (**c**) The heatmap represents the change in the expression levels of the genes and the accumulation levels of the metabolites. From red to blue or to green, the heatmap indicates the expression levels of genes or the accumulation levels of metabolites ranging from high to low.

To reveal the association between the fatty acid contents and gene transcription levels, we used all DEGs between the *Cn. tall* cultivar and each of the *Cn. dwarf* cultivars (YD vs. HT, GD vs. HT) for WGCNA analysis. A total of 12 expression modules were identified with turquoise modules containing the most genes and grey modules the least (Figure S5 and Table S8). Since the coefficient of variation results indicated that 50% of the polyamines had CV values greater than 1 (Figure 1c and Table S2), we investigated the co-relationship between the 12 expression modules and metabolite contents in the polyamine pathway using Pearson correlation coefficients. The correlation calculation results indicated that all seven polyamines in the spermine metabolism pathway were highly correlated with different modules (r > 0.5 or r < −0.5) except for “MEpurple”, “MEyellow”, and “MEgrey” (Figure 4a). The “MEpink” module showed the highest correlation coefficient of 0.96; therefore, we further analyzed the expression pattern of genes in the MEpink module. It was found that these genes were highly expressed in HT and expressed at a lower level in the *Cn. dwarf* cultivars (Figure 4b). Due to metabolome data showing that the content of ferulic spermidine in HT was much higher than that in GD and YD, we speculate that these modules may contain genes related to ferulic spermidine biosynthesis.

Based on metabolome quantification and RNA-seq results, we mapped the coconut polyamine metabolic pathway. We found that ferulic acid precursor metabolites, such as coumaric acid (Cm021), caffeic acid (Cm027), and ferulic spermidine (Cm142), accumulated specifically in HT, while other polyamine metabolites were highly accumulated in GD. To explore the molecular mechanism of the ferulic acid biosynthesis, we detected the gene expression levels in the ferulic acid biosynthesis pathway. We found that the ferulic acid metabolism genes 4CL and SHT have multiple homologs in coconut, and all homologs had high expression levels in HT and were present in the “MEpink” module. On the other hand, we found that the downstream biosynthetic gene COMT was highly expressed in GD, which was consistent with the pattern of metabolite accumulation (Figure 4c).

### 3.5. WGCNA Reveals Regulatory Networks Related to α-Linolenate

**Figure 5:**
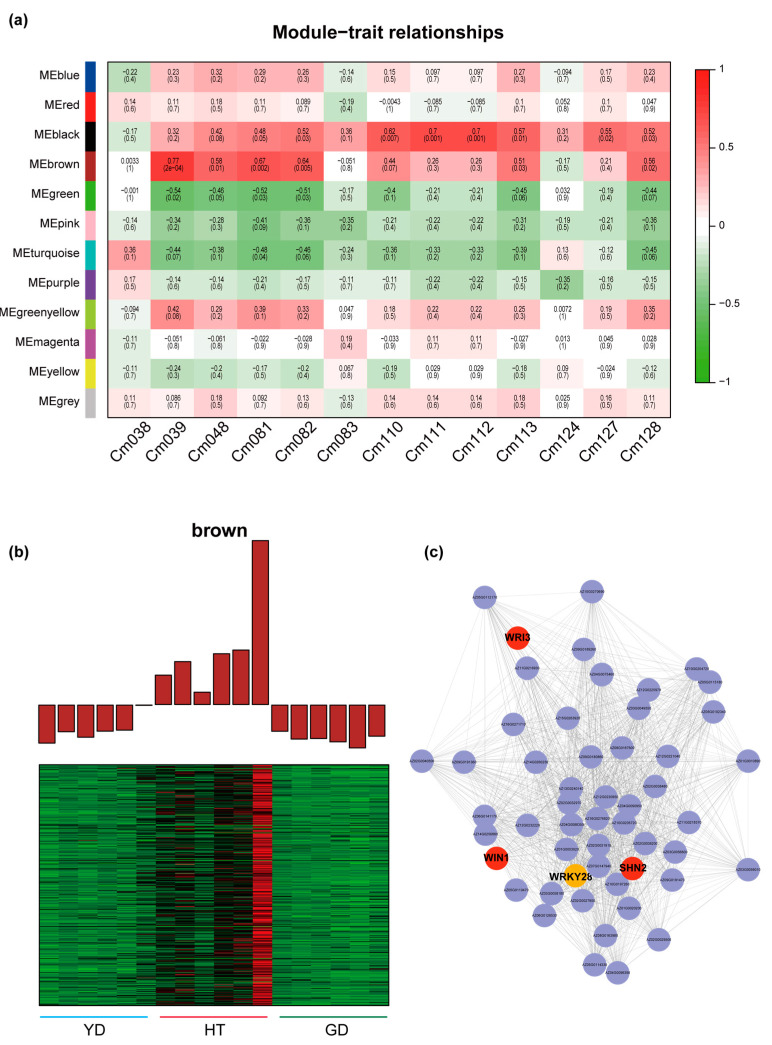
Exploring the regulators involved in the biosynthesis of α-linolenate and related metabolites with WGCNA. (**a**) Heatmap of the correlation between module genes and fatty acids: each row represents a module, each column represents a fatty acid, and the color and value of each cell at the intersection of the row and column represent the correlation coefficient between the module and the sample and *p*-value. Cm038: (9Z)-12-Oxo-dodec-9-enoate; Cm039: Traumain; Cm048: Trau-matic acid; Cm081: γ-Linolenic acid; Cm082: α-Linolenate; Cm083: Linoleic acid; Cm110: 12-OPDA; Cm111: 12,13(S)-EOTrE; Cm112: 9,10-EOTrE; Cm113: OPC8; Cm124: Di-homo-γ-linolenic acid; Cm127: 13(S)-HpOTrE; Cm128: 9-Hydroxy-12-oxo-15(Z)-octadecenoic acid. (**b**) Expression profiles of “MEbrown” modules. (**c**) Transcriptional regulatory network in different coconuts.

Since the *α*-linolenate content in *Cn.tall* and *Cn. dwarf* differed significantly, we performed correlation analysis between the contents of α-linolenate and several other lipids with WGCNA modules. Four modules were found to be highly correlated with the content of these metabolites (r > 0.5 or r < −0.5) (Figure 5a), implying that some genes belonging to these modules may play roles in α-linolenate acid and lipid biosynthesis.

Further profiling showed that two modules were significantly correlated with *α*-linolenate, namely “MEgreen” and “MEbrown”. The correlation between “MEbrown” and *α*-linolenate was the highest (r = 0.64, *p* = 0.005); therefore, we analyzed this module in depth. The module contains 618 genes and is highly expressed in HT but expressed at a much lower level in the two *Cn. dwarf* cultivars (Figure 5b). Further, we refined a sub-network in this module. It was found that there are several key genes in this network, such as *WRI3*, *WIN1,* and *SHN2*, which play crucial roles in plant lipid biosynthesis [33,34,35]. We then identified a transcription factor in this network, *WRKY28*, which had more than 50 linkages with other genes, indicating that *WRKY28* might be a “hub gene” in α-linolenate biosynthesis (Figure 5c).

**Figure 6:**
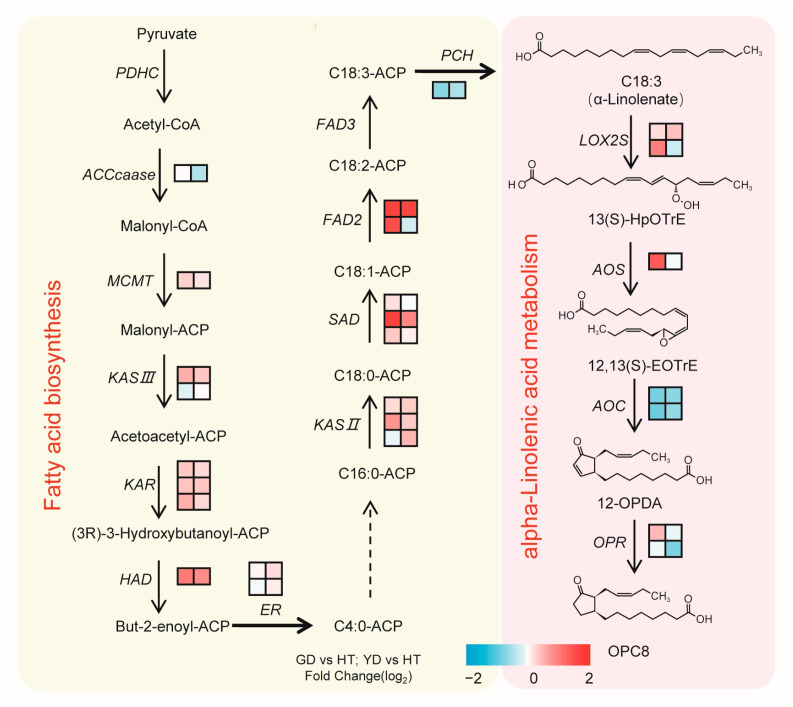
Synthetic pathway of coconut fatty acid. The pathway was constructed based on KEGG and the literature. The left color block to the right of each gene transcript level represents the log_2_ (fold change) between GD and HT. The right color block to the right of each gene transcript level represents the log_2_ (fold change) between YD and HT.

By integrating our metabolome and transcriptome data, we then reconstructed a α-linolenate biosynthetic pathway in coconuts (Figure 6). As Figure 6 shows, most of the genes that participated in α-linolenate biosynthesis, such as *FAD2*, *SAD*, and *KAS II*, exhibited higher transcription levels in GD and YD than HT. However, *PCH*, a key gene for the synthesis of α-linolenate, showed a higher transcription level in HT than GD and YD (Figure 6). *LOX2S* and *AOS* were two key genes in the metabolism of α-linolenate, and they were both highly expressed in the *Cn. dwarf* cultivars with higher expression in GD. However, *AOC*, another key gene involved in *α*-linolenate metabolism, was expressed at a higher level in HT. These results indicated a divergent regulatory mechanism in α-linolenate biosynthesis between the *Cn. dwarf* and *Cn. tall* cultivars.

## 4. Discussion and Conclusions

Although some metabolomics studies have been conducted on coconut, the identification of its nutritional components is still incomplete. In a previous study, only a small variety of metabolites were identified in coconut water, while a large number of amino acids and their derivatives were identified in flesh [8]. In accordance with this result, we identified 52 amino acids in coconut flesh. In addition, 8 polyamines and 158 lipids were newly identified in this work (Figure 1b). Our work has enriched our knowledge of the diversity of coconut nutrients, especially in amino acids, polyamines, and lipids.

Polyamines are not only beneficial for the human body but also crucial for plant development [2,32,36]. We found higher accumulation of polyamines in HT, and a gene module containing *4CL*, *SHT* was found to be highly expressed in HT in the WGCNA analysis (Figure 4a,b). These results imply that DEGs in the module are responsible for the higher polyamine contents of HT. Polyamines can promote cell growth and differentiation, embryo formation, and fruit development and ripening, and they also participate in various stress responses. For instance, caffeic acid and coumaric acid play roles in resisting abiotic stresses, such as heavy metal and salt stress [37,38,39,40,41]. Considering that *Cn. tall* is more tolerant of growing by the sea than *Cn. dwarf*, the more abundant polyamines in *Cn. tall* seeds may help resist environments with high salinity and humidity. Thus, our study not only explored the nutritional diversity of coconut flesh but also revealed their differences in development and tolerance.

The strategy of combining transcriptome and metabolome analysis can help clarify the molecular mechanism of metabolic variation. Glutamate is an important precursor metabolite of glutathione, and *astC* is an important gene for glutamate synthesis [42]. Our results showed that the expression pattern of *astC* was consistent with the accumulation pattern of glutamate, indicating that *astC* might also be involved in glutamate biosynthesis in coconuts. Additionally, *WRKY28* was found co-expressed with several lipid related genes, including *WRI3*, *WIN1*, and *SHN2*. *WRI3* and *WIN1* function as catalysts in lipid biosynthesis in plants, and *SHN2* is involved in the regulation of the synthesis of the plant cuticle [43]. Therefore, we speculate that *WRKY28* may regulate the expression of *WRI3*, *WIN1, SHN2*, or other genes to regulate lipid metabolism in *Arabidopsis thaliana*; *WRKY* family transcription factor *WRKY33* and *WRKY6* are associated with lipid biosynthesis [44,45]. Due to the proven role of *WRKY28* in regulating salicylate biosynthesis, the study of the relationship between lipid and SA biosynthesis is also interesting [46].

In this study, we identified a number of new nutrients, including amino acids and their derivatives, polyamines, and lipids, in coconut. By integrating metabolomic and transcriptomic analyses, we found that the differential expression of several key genes was responsible for the metabolic variation in different coconut cultivars. A novel transcription factor gene, *WRKY28*, was suggested to participate in the regulatory network of lipid biosynthesis. Our research has broadened our understanding of the nutritional quality of coconut and provided clues for further research on its molecular mechanism.

## Data Availability

All data and materials are available on request. RNA-seq data can be found below: National Center for Biotechnology Information (NCBI) BioProject database under accession number was PRJNA937045.

## Supplementary Materials

The following supporting information can be downloaded at: https://www.mdpi.com/article/10.3390/metabo13060683/s1, Figure S1: Identification of metabolites by mass spectrometry; Figure S2: OPLS-DA model diagram of LC–MS metabolomes of three cultivars of coconut; Figure S3: The number of differential metabolites among different coconut cultivars; Figure S4: PCA of RNA-seq data; Figure S5: Hierarchical clustering tree (dendrogram) of genes based on co-expression network analysis; Figure S6: GO analysis of differential genes between GD and YD; Figure S7: GO analysis of differential genes between HT and YD; Figure S8: GO analysis of differential genes between GD and HT. Table S1: Raw metabolic data; Table S2: Metabolites content in samples; Table S3: Statistics of differential accumulation of metabolites; Table S4: Quality control information for RNA-seq data; Table S5: TPM value for each gene in RNA-seq data; Table S6: Differentially expressed genes in HT vs. YD; Table S7: Differentially expressed genes in HT vs. GD; Table S8: Differentially expressed genes in GD vs. YD; Table S9: Summary of modules in WGCNA.
