# Supplementary material for: Comprehensive Analysis of Metabolome and Transcriptome Reveals the Regulatory Network of Coconut Nutrients
Source: Metabolites. 2023 May 24;13(6):683. doi: 10.3390/metabo13060683 (PMC10302879; doi:10.3390/metabo13060683)
Supplement: Supplementary file 1 [file metabolites-13-00683-s001.zip › metabolites-2307344-supplementary/Supplementary Informations/Figures/Figure S5.pdf]

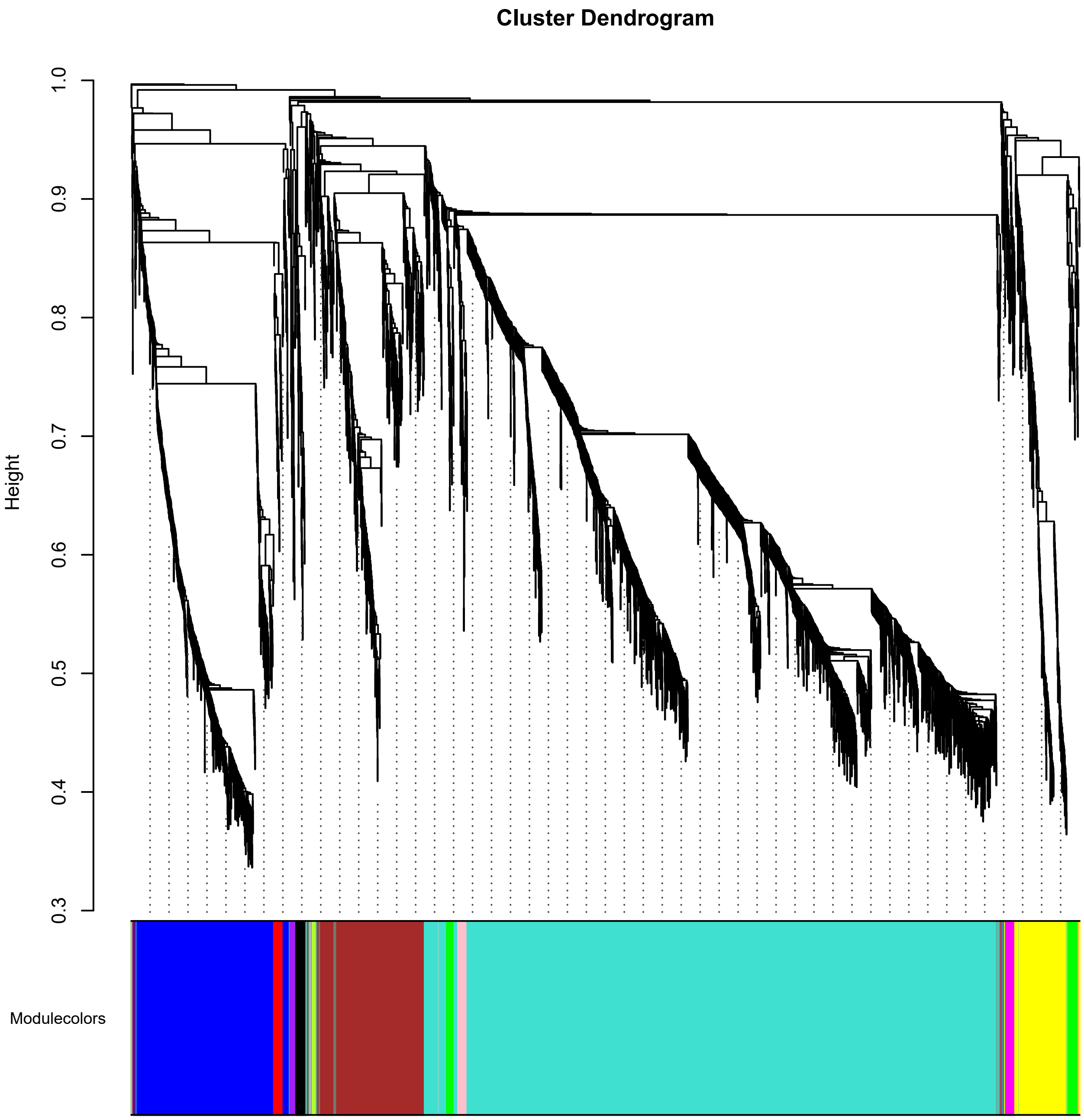

Figure S5: Hierarchical clustering tree (dendrogram) of genes based on coexpression network analysis.
