# Supplementary figures and images for: Comprehensive Analysis of Metabolome and Transcriptome Reveals the Regulatory Network of Coconut Nutrients
Source: Metabolites. 2023 May 24;13(6):683. doi: 10.3390/metabo13060683 (PMC10302879; doi:10.3390/metabo13060683)

**(a)**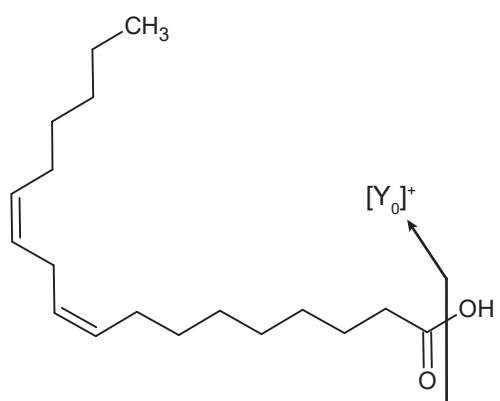**(b)**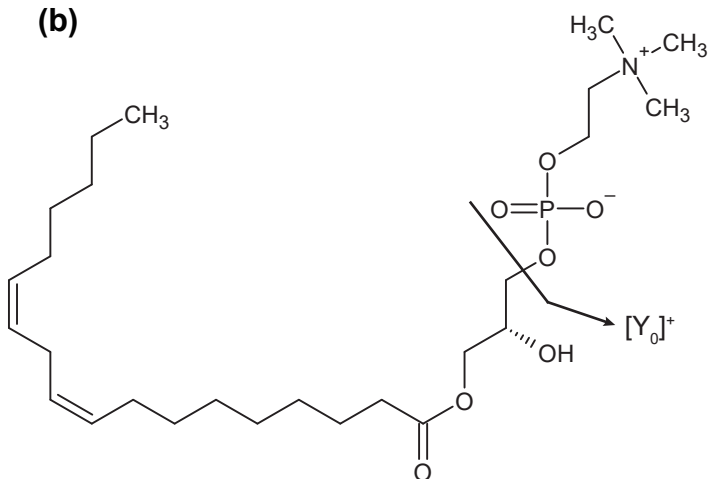**(c)**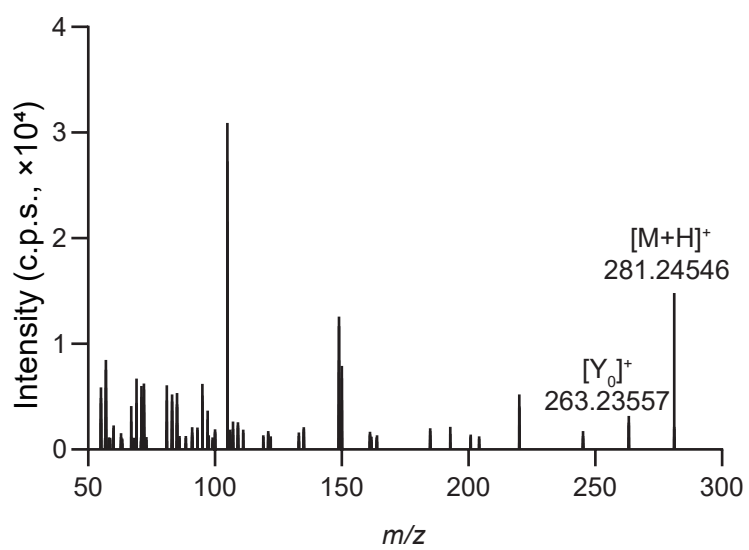**(d)**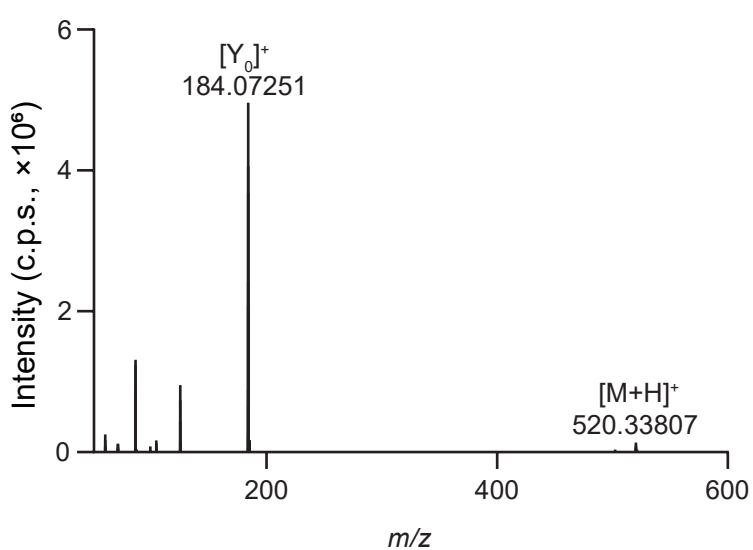

Figure S1: Identification of metabolites by mass spectrometry.

Supplement: Supplementary file 1 [file metabolites-13-00683-s001.zip › metabolites-2307344-supplementary/Supplementary Informations/Figures/Figure S1.pdf]

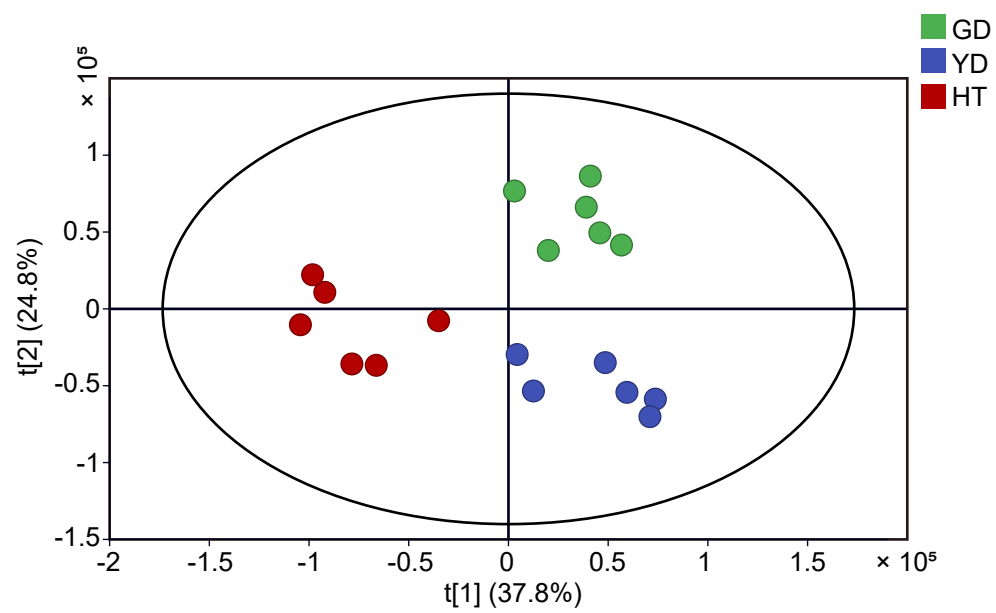

Figure S2: OPLS-DA model diagram of LC-MS metabolomes of three cultivars of coconut.

Supplement: Supplementary file 1 [file metabolites-13-00683-s001.zip › metabolites-2307344-supplementary/Supplementary Informations/Figures/Figure S2.pdf]

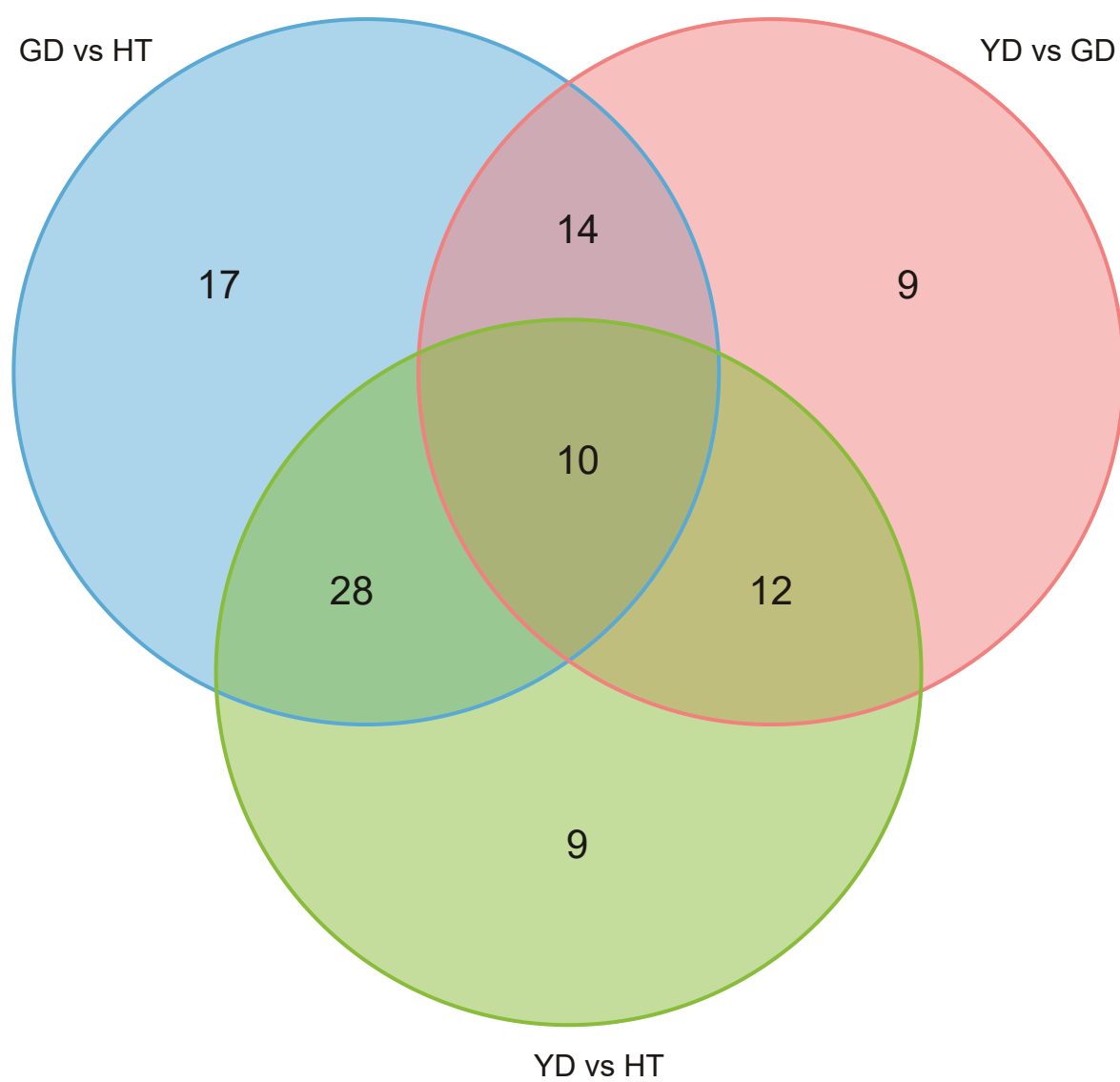

Figure S3: The number of differential metabolites among different coconut cultivars.

Supplement: Supplementary file 1 [file metabolites-13-00683-s001.zip › metabolites-2307344-supplementary/Supplementary Informations/Figures/Figure S3.pdf]

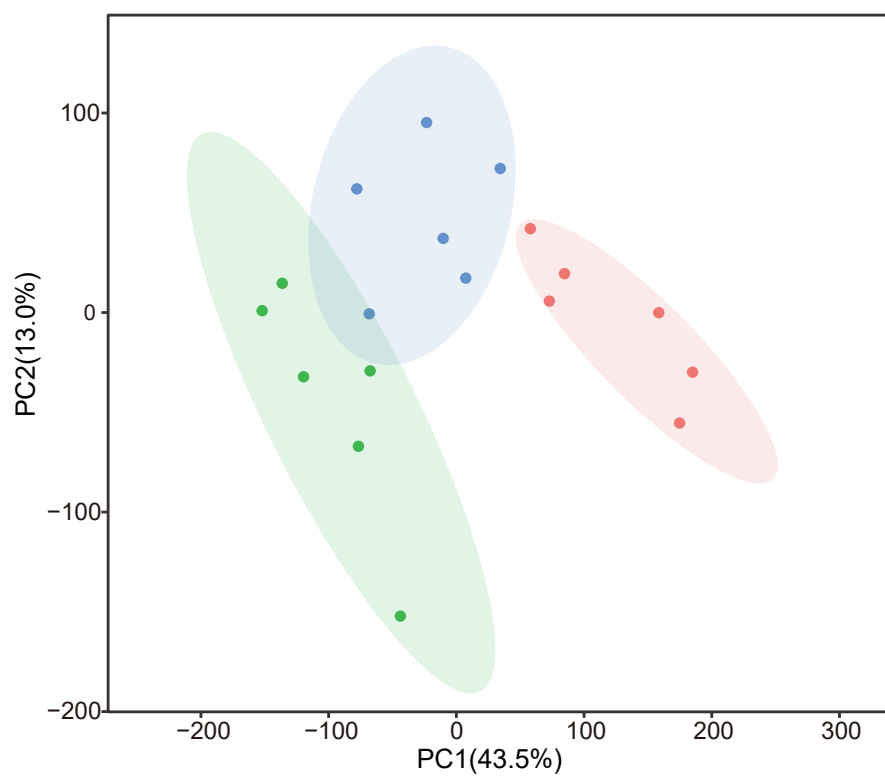

Figure S4: PCA of RNA-seq data.

Supplement: Supplementary file 1 [file metabolites-13-00683-s001.zip › metabolites-2307344-supplementary/Supplementary Informations/Figures/Figure S4.pdf]

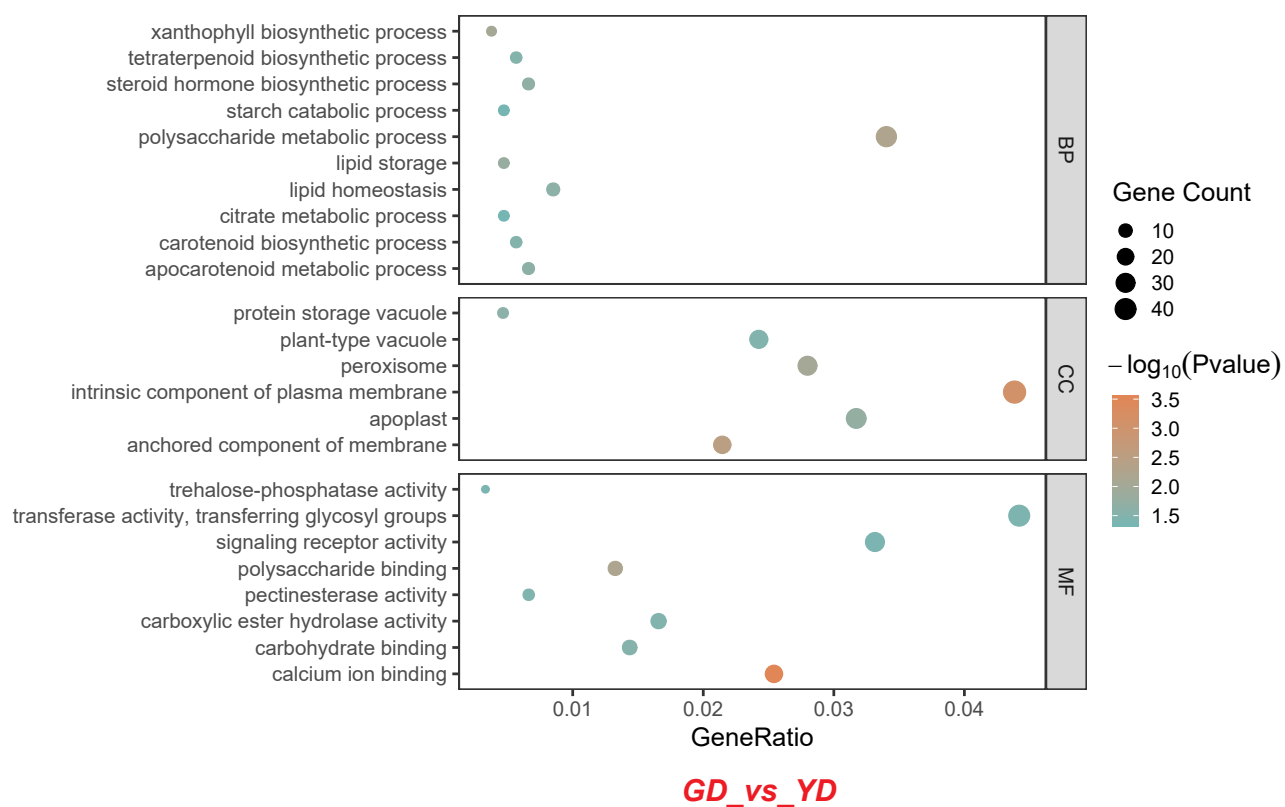

Figure S6: GO analysis of differential genes between GD and YD.

Supplement: Supplementary file 1 [file metabolites-13-00683-s001.zip › metabolites-2307344-supplementary/Supplementary Informations/Figures/Figure S6.pdf]

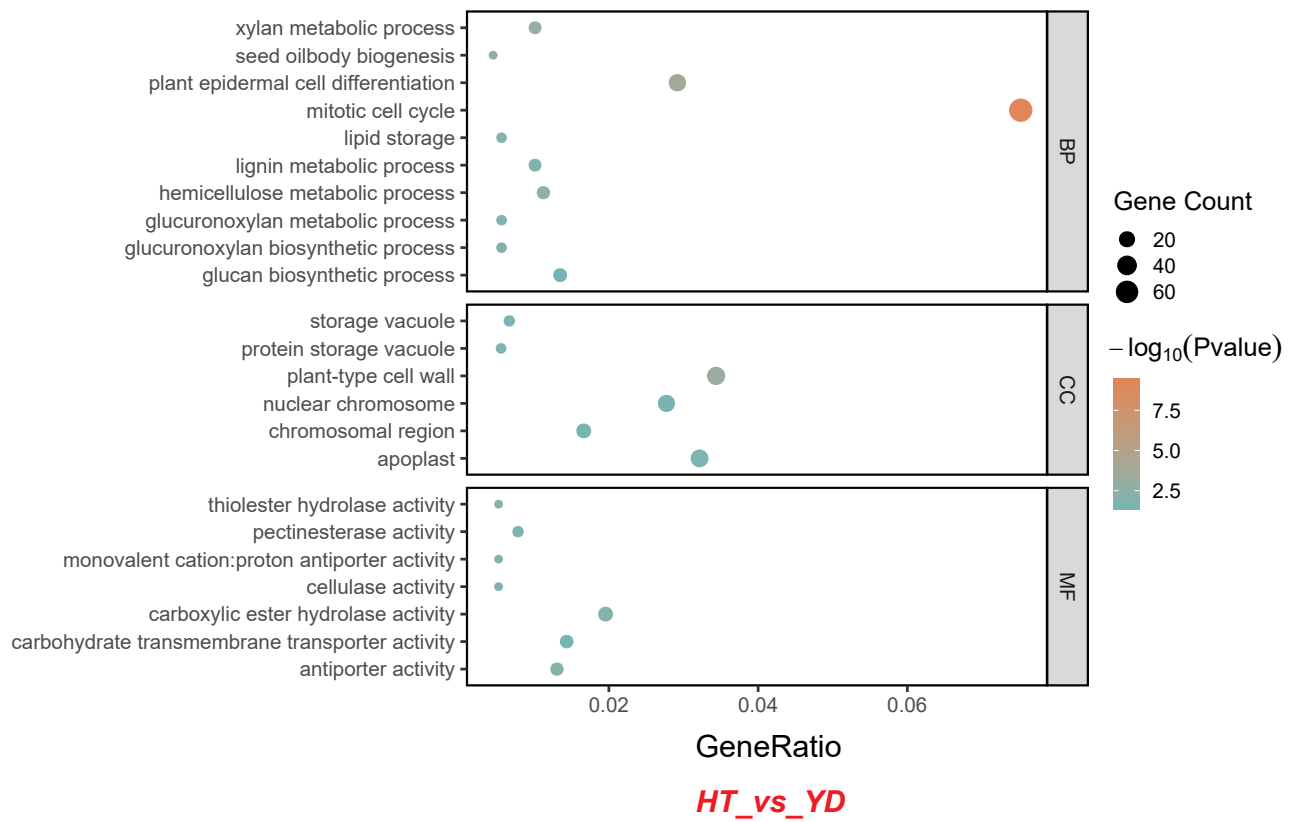

Figure S7: GO analysis of differential genes between HT and YD.

Supplement: Supplementary file 1 [file metabolites-13-00683-s001.zip › metabolites-2307344-supplementary/Supplementary Informations/Figures/Figure S7.pdf]

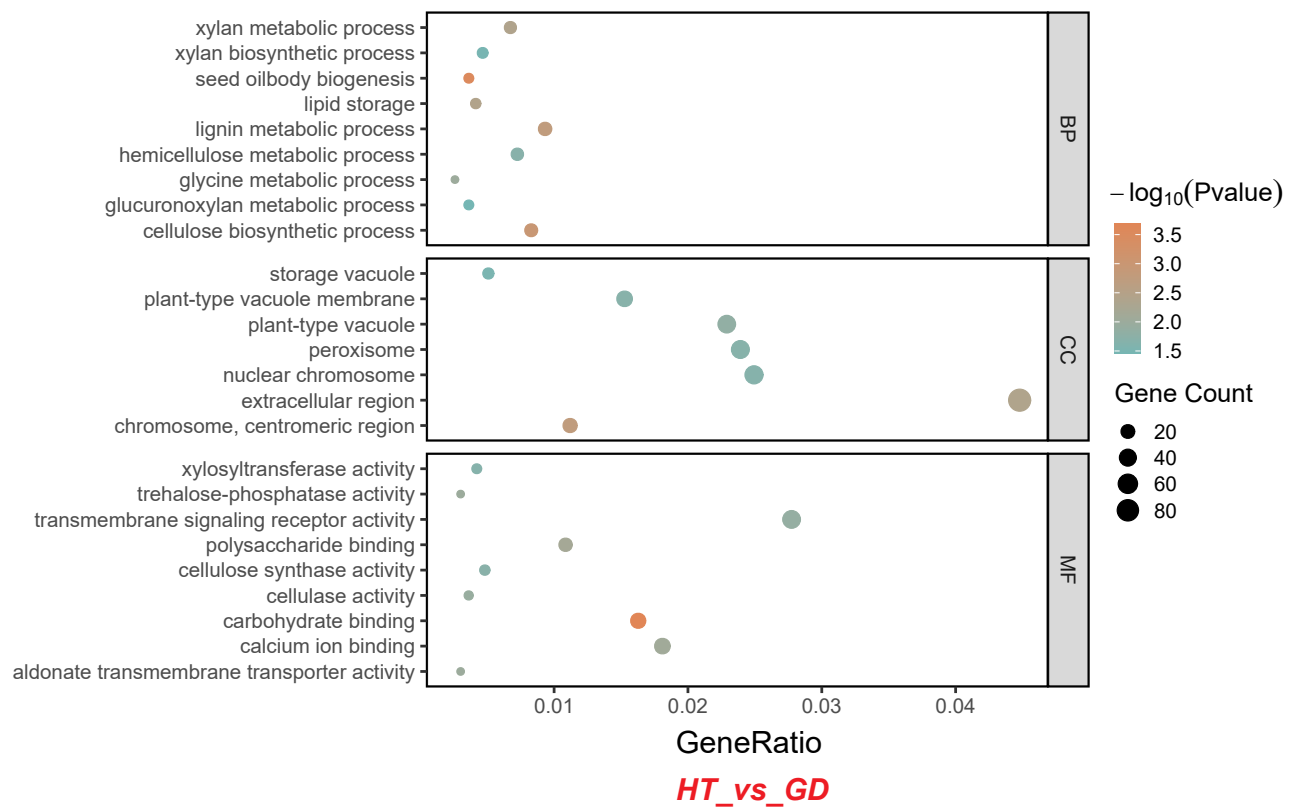

Figure S8: GO analysis of differential genes between GD and HT.

Supplement: Supplementary file 1 [file metabolites-13-00683-s001.zip › metabolites-2307344-supplementary/Supplementary Informations/Figures/Figure S8.pdf]
